# Long‐Term Clinical and Radiographic Outcomes of Large Periapical Lesions Managed by Non‐Surgical and Surgical Endodontic Treatment: A Case Series

**DOI:** 10.1002/ccr3.73727

**Published:** 2026-10-11

**Authors:** Mohsen Aminsobhani, Maryam Babaahmadi, Razieh Amiri, Maryam Habibzadeh, Nazanin Kamali

**Affiliations:** ^1^ Department of Endodontics, Dental Research Center AJA and Tehran University of Medical Sciences Tehran Iran; ^2^ Department of Endodontics, School of Dentistry Tehran University of Medical Sciences Tehran Iran

**Keywords:** apical periodontitis, large periapical lesion, long‐term follow‐up, mineral trioxide aggregate, non‐surgical endodontic treatment, periradicular surgery

## Abstract

The management of large periapical lesions is challenging, as lesion size alone does not predict treatment outcome or mandate surgery. This case series presents three large periapical lesions with 4‐ to 12‐year follow‐up, illustrating different therapeutic pathways. Two cases (mandibular canine/premolar and maxillary lateral incisor) resolved with non‐surgical treatment, achieving radiographic healing at 12‐year and 5‐year follow‐ups, respectively; the lesion in Case 1 persisted at 2 years but resolved by 12 years. The third case (maxillary lateral incisor with sinus tract and swelling) failed non‐surgical retreatment, requiring periradicular surgery with MTA, resulting in complete healing at 4 years. All cases achieved Periapical Index scores of 2. This series demonstrates that non‐surgical management remains first‐line, but persistent symptoms, sinus tracts, or lack of radiographic improvement at 1 year should prompt surgery. Long‐term follow‐up is essential to distinguish delayed healing from true failure.

## Key Clinical Message

Large periapical lesions may heal with non‐surgical treatment, though healing can be delayed for years. Persistent symptoms or drainage at 1‐year warrant reassessment. Surgery may be indicated when clinical signs of infection persist or non‐surgical management is unlikely to resolve the pathology.

## 1 Introduction

Managing large periapical lesions is challenging: while non‐surgical treatment is first‐line, its success rate diminishes with lesion size, creating uncertainty about when to transition to surgery [1, 2]. Pulpal necrosis from caries or trauma allows microorganisms to invade the root canal, initiating apical periodontitis [3]. Although canal microbial diversity is limited, interspecies synergy is a major virulence determinant for larger lesions [3, 4]. Bacterial antigens provoke a host inflammatory response, yet pathogens employ evasion strategies [3, 5].

Over 90% of periapical lesions are granulomas, radicular cysts, or abscesses [6]. Radicular cysts typically arise from Malassez's cell rests [7]. However, conventional imaging, including CBCT, cannot reliably differentiate granulomas from cysts, making histopathology the diagnostic gold standard [3, 8]. Cystic lesions are suggested by size > 200 mm^2^, non‐vital teeth, well‐defined borders, and straw‐colored fluid [1]. Cyst‐like lesions are associated with intra‐ and extra‐radicular infections, show no pain, but demonstrate progressive radiographic enlargement [9]. Large lesions more frequently represent true cysts with an intact epithelial lining that may not respond to non‐surgical treatment [1].

Management options include non‐surgical endodontic therapy or, after failed conservative treatment, surgical approaches such as curettage, apicoectomy, marsupialization, or tube decompression [10, 11]. Strindberg's criteria define success as radiographic resolution and symptom absence [12]. While small granulomas often respond to non‐surgical treatment, Oztan (2002) showed that larger lesions, including cysts, respond well to calcium hydroxide, suggesting size alone should not dictate surgery [11]. When orthograde therapy fails, periapical surgery is a viable alternative [13]. MTA is the preferred root‐end filling material for its biocompatibility and sealing [1].

This case series presents three large periapical lesions managed through different approaches, illustrating the importance of individualized treatment planning, timely recognition of failure, and long‐term monitoring. Table 1 summarizes baseline clinical characteristics, radiographic findings, and treatment history for each case.

**TABLE 1:** Baseline clinical characteristics and treatment history.

Parameter	Case 1	Case 2	Case 3
Tooth	#43, #44	#12	#22
Age/Sex	40/F	30/F	42/F
Medical status	ASA I	ASA I	ASA I
Lesion dimensions (mm)	20 *15	10 * 13	10 * 7
Periodontal probing (mm)	2–3	2–3	2–4
Tooth mobility	0	Grade I	0
Percussion	Severe (#43), Mild (#44)	Severe	Marked
Palpation	NR	Tender	Tender
Sinus tract	No	No	Yes
Previous RCT	#43 treated	None	#22 treated
Coronal restoration	Incomplete	Composite	Incomplete
Cracks/fractures	None	None	None
Root resorption	None	None	None
CBCT available	No	No	No

## 2 Case Presentation

### 2.1 Case 1

A 40‐year‐old female (ASA I) presented with pain on eating in the right mandible. Intraorally, teeth #43 and #44 were non‐responsive to cold testing; #43 showed severe and #44 mild percussion tenderness. Radiography revealed incomplete root canal treatment of #43 and a large, well‐circumscribed periapical radiolucent lesion surrounding the roots of both teeth, measuring ~20 mm mesiodistally and 15 mm vertically (Figure 1A). CBCT was unavailable. Tooth #43 was diagnosed with Previously Treated Root Canal with Symptomatic Apical Periodontitis, and tooth #44 with Pulpal Necrosis with Symptomatic Apical Periodontitis. Retreatment of #43 and primary treatment of #44 were planned. After anesthesia (2% lidocaine with 1:80,000 epinephrine), access cavities were prepared. Retreatment of #43 used chloroform and rotary files. Working length was determined electronically. Canal preparation used crown‐down technique with Profile instruments to apical size 35, copiously irrigated with 5.25% NaOCl, saline, and 17% EDTA. Ultrasonic activation (CaviEndo, setting 6, 5 min/canal) was performed with the tip 2–3 mm short of working length. A calcium hydroxide/chlorhexidine medicament was placed. At 7 days, symptoms resolved. Canals were irrigated and obturated with lateral compaction using gutta‐percha and AH‐26 sealer (Figure 1B). At 2‐year follow‐up, the lesion was radiographically unchanged, though the patient was asymptomatic (Figure 1C). The patient was subsequently lost to regular recall but maintained occasional phone contact, consistently reporting no symptoms. She re‐presented 12 years later. Clinical examination confirmed the tooth was asymptomatic with normal mobility, negative percussion, and stable probing depths. Radiography demonstrated complete resolution with new bone formation (Figure 1D). The absence of intermediate radiographs between 2 and 12 years is acknowledged as a limitation.

**FIGURE 1:**
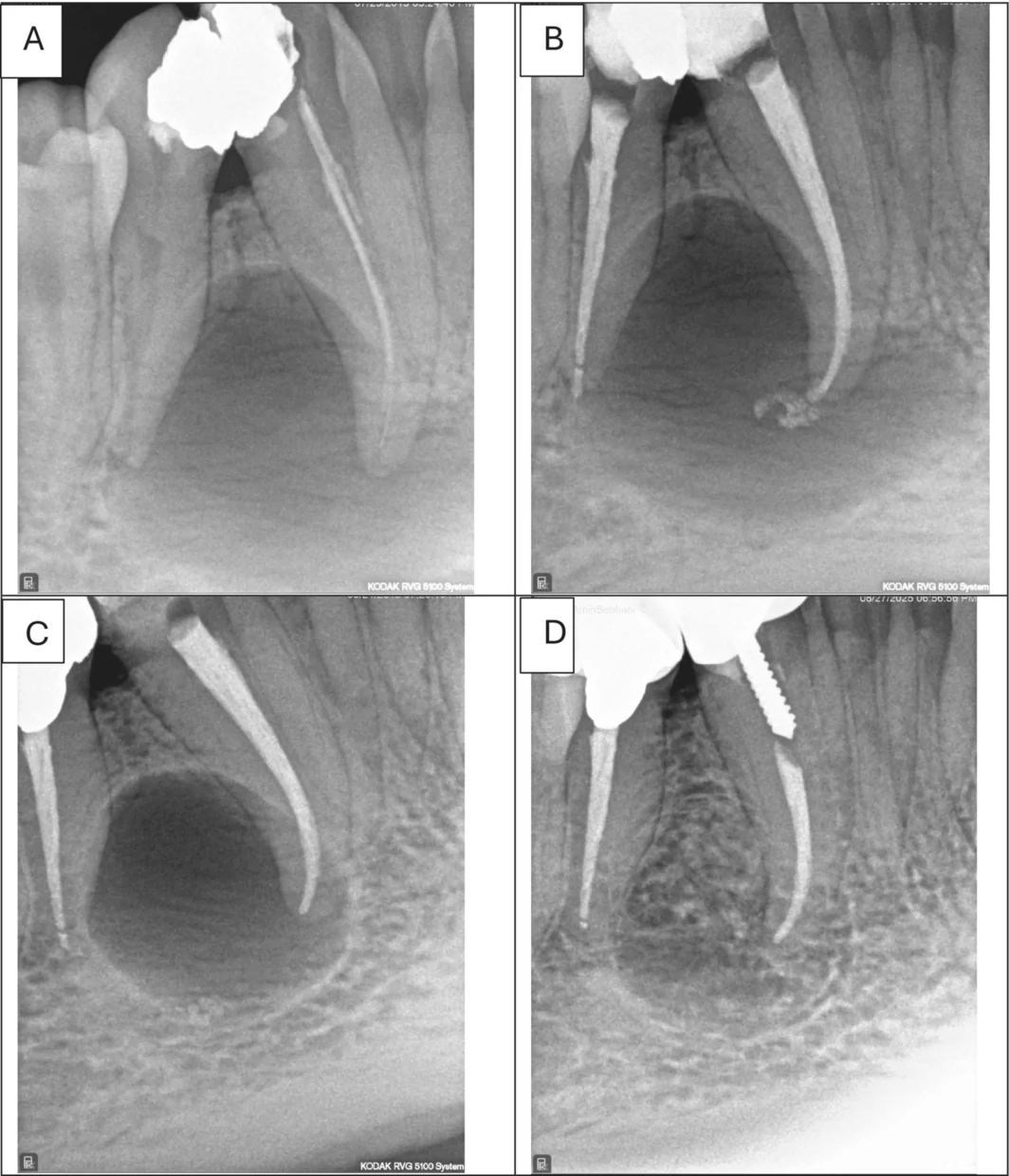
(A) Initial radiograph: Incomplete root canal treatment of tooth #43 and a large, well‐circumscribed periapical radiolucent lesion involving teeth #43 and #44; (B) Post‐treatment radiograph: Obturation of both canals using lateral compaction technique with gutta‐percha and AH‐26 sealer; (C) 2‐year follow‐up: Persistent circular radiolucent lesion with well‐defined borders and no significant size reduction; patient asymptomatic; (D) 12‐year follow‐up: Complete bone regeneration and total resolution of the periapical radiolucency, indicating satisfactory healing.

### 2.2 Case 2

A 30‐year‐old female presented with periodic swelling and pus drainage from the maxillary anterior region. Tooth #12 was tender to percussion and palpation, non‐responsive to cold/electrical testing, with Grade I mobility but no pockets. Radiography revealed a large, unilocular, well‐defined radiolucency surrounding #12, measuring ~10 × 13 mm (Figure 2A). CBCT was unavailable. Diagnosis: Pulpal Necrosis with Symptomatic Apical Periodontitis. After anesthesia and rubber dam isolation, access yielded suppurative drainage. Working length was determined, and canals were prepared using crown‐down technique with #15–40 Flexo‐Files, irrigated with 2.5% NaOCl and saline with sonic activation. Calcium hydroxide paste was placed. Root canal preparation was completed over two visits; obturation criteria included absence of symptoms, no exudate, and dry paper points. At 12 days, the tooth was asymptomatic. Medicament was removed, and obturation was performed with lateral condensation using gutta‐percha and AH Plus sealer (Figure 2B). At 5‐year follow‐up, clinical evaluation was normal, and radiographs demonstrated substantial reduction of the radiolucency with progressive bone regeneration (Figure 2C).

**FIGURE 2:**
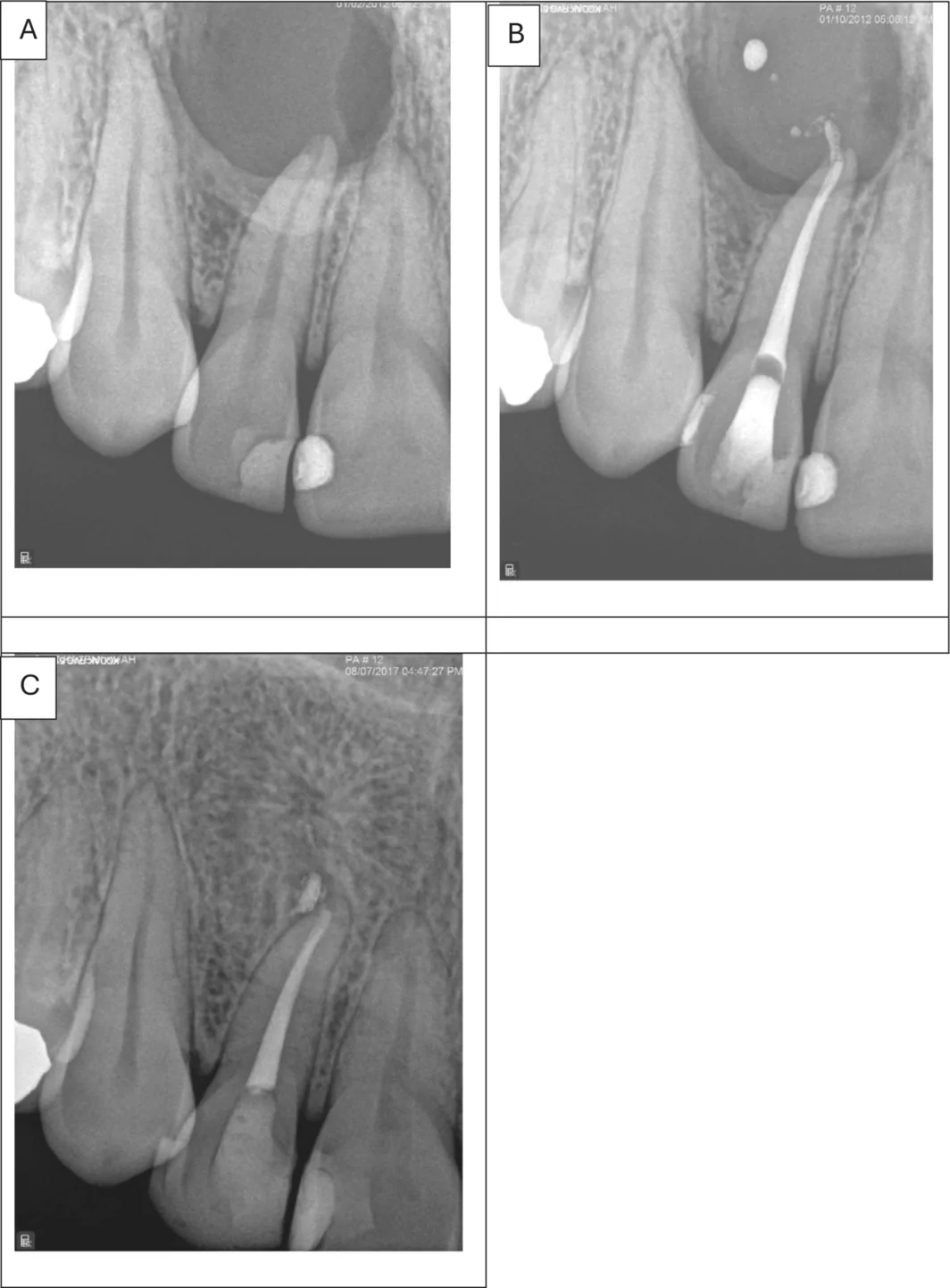
(A) Preoperative periapical radiograph demonstrating a large, unilocular, well‐defined radiolucent lesion surrounding the root of the maxillary lateral incisor (tooth #12), consistent with an extensive periapical lesion of endodontic origin; (B) Post‐obturation periapical radiograph following root canal therapy of tooth #12, showing obturation performed using the lateral condensation technique with gutta‐percha and AH Plus sealer; (C) Periapical radiograph obtained at the 5‐year follow‐up, revealing evidence of progressive bone regeneration and significant resolution of the periapical radiolucency, indicative of a favorable healing response.

### 2.3 Case 3

A 42‐year‐old female (ASA I) presented with a 6‐month history of pain, swelling, and purulent exudate in the left anterior maxilla. Extraoral examination revealed facial asymmetry and diffuse edema (30 × 40 mm). Intraorally, vestibular swelling and a draining sinus tract were observed. Tooth #22 was non‐responsive to thermal/electrical testing. Radiography revealed a substantial periapical radiolucency associated with previously treated #22, measuring ~10 × 7 mm (Figure 3A). CBCT was unavailable. After informed consent, non‐surgical retreatment was initiated. Gutta‐percha was removed with chloroform and rotary instruments. Working length was determined radiographically (Figure 3B). Irrigation with saline and 5.25% NaOCl with ultrasonic activation was performed. Instrumentation used ProTaper files to apical size 30, and calcium hydroxide/chlorhexidine medicament was placed. A releasing incision facilitated drainage. At two weeks, the patient was asymptomatic with complete resolution of swelling. The canal was re‐irrigated. Obturation criteria included absence of symptoms, no exudate, negative odor, and dry paper points. Obturation used gutta‐percha and AH Plus sealer via lateral compaction (Figure 3C). At one‐year follow‐up, the patient returned with a two‐month history of purulent discharge and tenderness. Tooth #22 was tender to percussion, and radiography showed a persistent radiolucent lesion (Figure 3D). After informed consent, periradicular surgery was scheduled. Under local anesthesia, a full‐thickness flap was elevated from #21 to #23. A buccal fenestration exposed the pathology. The apical 3 mm of #22 was resected, and root‐end cavity preparation was performed to 4 mm depth using ultrasonic retrotips. Retrograde filling used ProRoot MTA (Figure 3E). Periapical tissue was not submitted for histopathological examination; therefore, the precise pathological nature could not be determined. The flap was repositioned and sutured. Antibiotics (amoxicillin 500 mg TID for 5 days) and analgesics were prescribed as a precautionary measure based on institutional practice, though not strictly indicated by current guidelines [14]. The authors acknowledge that this prescription was based on institutional practice patterns rather than strict evidence‐based indications. At two‐week follow‐up, tissues appeared healthy. At four‐year follow‐up, complete bone healing with lamina dura re‐establishment was observed; the patient remained asymptomatic with no sinus tract recurrence (Figures 3F and 3G).

**FIGURE 3:**
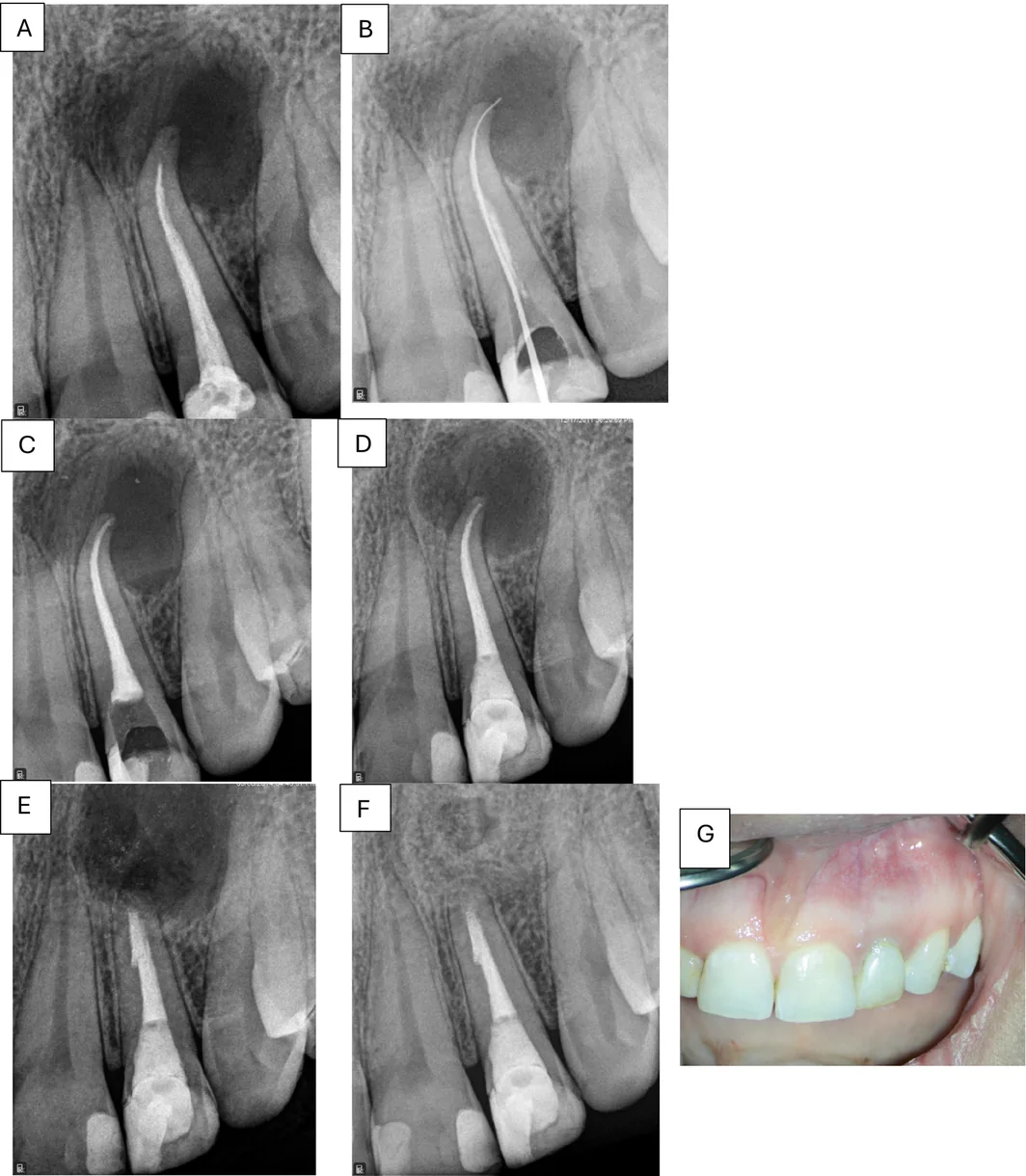
(A) Preoperative periapical radiograph showing a substantial periapical radiolucent lesion associated with the left maxillary lateral incisor (tooth #22), which had previously undergone endodontic treatment; (B) Working length determination radiograph obtained during the initial retreatment appointment, confirming the established working length prior to instrumentation; (C) Post‐obturation radiograph following non‐surgical retreatment of tooth #22, showing obturation using the lateral compaction technique with gutta‐percha and AH Plus sealer; (D) Periapical radiograph obtained at the 1‐year follow‐up, demonstrating a persistent radiolucent lesion with no appreciable change in size, correlating with the patient's recurrent clinical symptoms of purulent discharge and tenderness; (E) Immediate postoperative radiograph following periradicular surgery, confirming the placement of the retrograde filling material (ProRoot MTA) at the resected root end of tooth #22; (F) Periapical radiograph obtained at the 4‐year follow‐up, demonstrating complete bone healing and re‐establishment of the lamina dura, with no evidence of residual pathology; (G) Clinical photograph obtained at the 4‐year follow‐up, showing healthy periodontal tissues with no signs of inflammation, swelling, or sinus tract formation.

### 2.4 Radiographic Assessment

Lesion dimensions were estimated from standardized periapical radiographs using a radiographic ruler. The greatest mesiodistal and vertical diameters were recorded. CBCT was unavailable, limiting volumetric assessment. Two independent examiners evaluated periapical status using the PAI scoring system [15], assessing radiographs independently and resolving disagreements by consensus. PAI scores range from 1 to 5 (Table 2) [15, 16]. Healing was considered successful when the final PAI score decreased to 1 or 2; scores of 3–5 indicated persistent disease [16].

**TABLE 2:** Description of periapical index scores (adapted from Ørstavik et al. [15] and Penesis et al. [16]).

PAI score	Description of radiographic findings
1	Normal periapical structures
2	Small changes in bone structures
3	Change in bone structure with mineral loss
4	Periodontitis with well‐defined radiolucent area
5	Severe periodontitis with exacerbating features

All three cases showed improvement from preoperative score 4 to final score 2 (Table 3).

**TABLE 3:** Preoperative and Final Follow‐Up PAI Scores.

Case	Tooth	Preoperative PAI score	Final follow‐up PAI Score	Treatment modality	Follow‐up duration
1	#43	4	2	Non‐surgical re‐RCT	12‐Year
	#44	4	2	Non‐surgical RCT	12‐Year
2	#12	4	2	Non‐surgical RCT	5‐Year
3	#22	4	2	Non‐surgical re‐RCT + Apicoectomy	4‐Year

## 3 Discussion

Direct comparative studies of surgical versus non‐surgical management are scarce, with most reviews relying on indirect comparisons [17, 18, 19]. Success rates of endodontic surgery decline more over time than non‐surgical treatment [20, 21], but comparisons are confounded by baseline disparities [22]. This case series provides long‐term follow‐up of three large lesions, illustrating that non‐surgical therapy succeeds in many cases, but timely transition to surgery remains critical for refractory lesions.

Chronic periapical inflammation from bacterial invasion leads to radiolucent bone lesions [1]. These lesions act as host‐mediated barriers [23, 24]. Less frequently, an epithelial plug forms at the apical foramen to block extra‐radicular penetration [24]. Radiographically well‐defined lesions > 200 mm^2^ associated with non‐vital teeth that yield straw‐colored fluid are nearly 100% diagnostic of radicular cysts [1].

Most periapical lesions heal following non‐surgical treatment [24, 25]. However, healing is protracted: 50% show signs at 6 months, 88% at 12 months, and resolution may take up to 4 years [26]. A minimum 12‐month follow‐up is recommended before considering such teeth as abutments [24]. Delaying coronal restoration increases fracture risk, whereas a sound restoration promotes healing [24, 27]. Radiographic lesions alone should not trigger retreatment in asymptomatic well‐treated teeth, as flare‐up incidence is below 6% over 20 years [28]. Sound coronal restoration should be placed immediately [24]. Our cases illustrate these principles. In Case 1, the lesion showed no substantial reduction at 2 years but had completely resolved by the 12‐year examination. The absence of intermediate radiographic documentation between these time points means the precise timeline of healing cannot be determined; the lesion persisted at 2 years and healing was confirmed at 12 years. Conversely, in Case 3, the lesion remained symptomatic and active at 1 year, prompting timely surgery. These observations underscore highly variable healing timelines and the need for long‐term follow‐up.

Periradicular lesions are infectious entities requiring effective antimicrobial strategies for optimal outcomes [24]. Disinfection was achieved using NaOCl for its antimicrobial and tissue‐dissolving properties [29], supplemented with chlorhexidine and calcium hydroxide dressings [24]. All cases received antimicrobial activation (ultrasonic in Cases 1 and 3; sonic in Case 2) to enhance irrigation and biofilm disruption. In Case 3, persistent infection despite adequate disinfection required surgery.

Endodontic failures primarily arise from persistent intraradicular infections, necessitating retreatment before surgery [17, 30]. However, persistent radiolucencies may stem from extraradicular factors, including infections, cysts, cholesterol crystals, or scar tissue [24, 31, 32, 33]. Cases 1 and 2 resolved non‐surgically, underscoring meticulous debridement. Case 3 failed to respond, necessitating apical surgeryThe failure may reflect extraradicular infection, cholesterol crystals, or a true cystic lesion—factors not resolvable through intracanal therapy alone, though without histopathology, these explanations remain speculative. Persistent symptoms or drainage should prompt reassessment. Surgical intervention may be considered when persistent disease is accompanied by clinical signs of infection or when non‐surgical management is unlikely to resolve the problem.

Comparative analysis reveals clinically relevant distinctions. Cases 1 and 2 resolved non‐surgically (final PAI 2 at 12 and 5 years). Case 3 showed persistent disease at 1 year, requiring surgery before achieving a favorable outcome (final PAI 2 at 4 years). Several factors may explain these divergent outcomes [1], the sinus tract and purulent exudate in Case 3 suggest extraradicular infection or a true radicular cyst, both refractory to non‐surgical therapy [32, 34]. Notably, Cases 1 and 2 presented without sinus tracts, suggesting that the infection remained largely confined to the intraradicular space, which is more amenable to orthograde debridement [2], the larger extraoral swelling (30 × 40 mm) in Case 3 suggests a more extensive inflammatory response and greater likelihood of cystic degeneration [1], the previously treated canal in Case 3 may have contributed to microbial persistence due to complex anatomy, isthmuses, or residual biofilm [31], anatomical location may matter: maxillary lateral incisors (as in Case 3) are prone to variations like dens invaginatus or palatogingival grooves that can harbor persistent infection [35].

Resin‐based sealers (AH 26/AH Plus) were used. While well‐tolerated, their slow resorption may contribute to delayed healing [36]. The successful resolution in Cases 1 and 2 likely reflects thorough debridement, effective medication, and favorable host response rather than sealer properties alone. Comparative studies show no significant long‐term outcome differences among sealer types [36, 37, 38].

Case 3 fulfilled indications for periapical surgery: orthograde retreatment failed due to persistent infection [35, 39]; retreatment was impractical; and the lesion suggested a true cyst, cholesterol crystals, or extraradicular infection—factors not resolvable non‐surgically.

MTA was selected for retrograde filling following ultrasonic root‐end preparation due to its superior biocompatibility and sealing. Despite technique sensitivity, MTA offers predictable success [40].

Several limitations of this case series should be acknowledged. First, the small sample size (*n* = 3) precludes generalizable conclusions regarding treatment efficacy or superiority of any particular approach, as case series are inherently limited in their ability to establish causal relationships or predict treatment outcomes in broader populations. Second, the lack of histopathological confirmation for the periapical lesions, particularly in Case 3, limits our ability to definitively characterize the nature of the non‐healing lesion; while radiographic features may suggest certain pathologies, studies have demonstrated that conventional imaging, including CBCT, cannot reliably distinguish between periapical cysts and granulomas [8]. Third, the retrospective nature of the case review introduces potential selection and reporting biases, as the cases were not systematically collected from a predefined cohort. Fourth, the absence of standardized outcome assessments beyond radiographic evaluation (PAI score) and clinical symptoms limits the comprehensiveness of outcome measurement; validated patient‐reported outcome measures were not employed, and the PAI score, while useful for monitoring healing, has inherent limitations as a surrogate outcome [15]. Fifth, the use of conventional periapical radiographs obtained over long follow‐up periods presents inherent limitations. Radiographic projection geometry may vary between examinations despite efforts to standardize techniques, potentially affecting the comparability of serial images. Furthermore, two‐dimensional radiography cannot accurately assess the three‐dimensional volume of periapical lesions, and small changes in bone healing may not be detectable on conventional radiographs. These limitations should be considered when interpreting the radiographic outcomes presented in this series. Sixth, the management decisions were made on a case‐by‐case basis without randomization, introducing confounding variables that may have influenced outcomes, including differences in baseline lesion characteristics, prior treatment history, and patient‐specific factors. Finally, despite the strength of the long‐term follow‐up (4–12 years), which represents a notable advantage of this series, the small number of cases limits the ability to identify predictive factors for treatment success or failure. Future prospective studies with larger sample sizes, histopathological correlation, and standardized outcome measures would be valuable to validate these observational findings and to better elucidate the biological factors that distinguish lesions that respond to non‐surgical treatment from those requiring surgical intervention.

## 4 Conclusion

The fate of a large periapical lesion lies not in its size, but in its biology. Non‐surgical therapy remains the cornerstone, but sinus tracts, persistent symptoms, or failure to heal within one year should raise clinical suspicion of underlying extraradicular pathology—including possible true cysts, cholesterol crystals, or persistent biofilm—that may not be accessible to intracanal disinfection. However, in the absence of histopathological confirmation, these remain plausible hypotheses rather than established diagnoses. This series demonstrates that with meticulous treatment and vigilant follow‐up, both conservative and surgical pathways can succeed; the key is knowing when to transition. As our 12‐year Case 1 reminds us, healing is not always swift, but it is often worth the wait.

## Author Contributions

**Mohsen Aminsobhani:** conceptualization, methodology. **Maryam Babaahmadi:** conceptualization, writing – original draft. **Razieh Amiri:** investigation, validation. **Maryam Habibzadeh:** conceptualization, validation. **Nazanin Kamali:** investigation, validation.

## Funding

The authors have nothing to report.

## Ethics Statement

The Research Ethics Committee of Tehran University of Medical Sciences reviewed the study protocol and determined that formal ethical approval was not required for this case series, as it involved the retrospective presentation of clinical cases arising from routine clinical practice with no experimental interventions beyond standard care. Written informed consent was obtained from all three patients prior to the initiation of any treatment and again prior to the publication of this case series, in accordance with the journal's patient consent policy. Copies of the signed consent forms are retained by the corresponding author and are available for review upon request.

## Consent

Written informed consent was obtained from all three patients prior to treatment and again prior to publication. At initial consultation, treatment plans, alternatives, risks, and benefits were explained, and patients were informed of their right to withdraw. Separate consent for publication of de‐identified clinical data, radiographs, and photographs was obtained after follow‐up. Copies of signed consent forms are retained by the corresponding author.

## Conflicts of Interest

The authors declare no conflicts of interest.

## Data Availability

The data supporting the findings of the present study are available from the corresponding author upon request.
